# Digital Health Training, Attitudes and Intentions to Use It among Romanian Medical Students: A Study Performed during COVID-19 Pandemic

**DOI:** 10.3390/healthcare11121731

**Published:** 2023-06-13

**Authors:** Lucia Maria Lotrean, Simina Antonia Sabo

**Affiliations:** Department of Community Medicine, Iuliu Hatieganu University of Medicine and Pharmacy, 400347 Cluj-Napoca, Romania

**Keywords:** Romania, medical students, digital health, medical education

## Abstract

Introduction: This study focuses on medical students from the University of Medicine and Pharmacy in Cluj-Napoca, Romania, and has three objectives. First, it evaluates the opinions of medical students regarding their previous training as well as their needs for future training in the field of digital health. Second, it assesses their attitudes regarding digital health and their intention to use digital tools as physicians. Lastly, the interrelationship between these issues as well as the socio-demographic factors which influence them are investigated. Materials and methods: A cross-sectional survey was performed during June–August 2021 among fifth and sixth year students of the Faculty of Medicine from the Iuliu Hațieganu University of Medicine and Pharmacy in Cluj-Napoca, Romania. Anonymous online questionnaires were used which were filled in by 306 students. Results: Less than half of the participating students declared that they benefited from training or different practical examples during medical education regarding the use of digital tools in different medical areas, while the majority said that they would like to receive more training in the field of digital health. A total of 58.2% said that they totally agree with the introduction of a formal training in the medical curricula regarding digital health. Many students declared positive attitudes toward the use of digital tools in different domains within the medical field and intention to use digital tools as physicians; several differences were noted, including gender, year of study, type of domain, and previous training with regard to the use of digital tools in those domains. Moreover, the need for future training and the desire for the introduction of a formal training program into the medical curricula with regard to this field were stronger among those with more positive attitudes and higher intentions to use digital tools in their medical activity. Conclusions: To the best of our knowledge, this is the first study from Romania which investigated the training, attitudes, and intentions regarding the use of digital health among Romanian medical students, and offers valuable information to guide the education of medical students.

## 1. Introduction

“A Europe fit for the digital age” is one of the European Commission’s six policy priorities for 2019–2024, and health is one of the sectors on this agenda [1]. The World Health Organization defines digital health as the “field of knowledge and practice associated with the development and use of digital technologies to improve health”, underlining that they “expand the concept of eHealth to include digital consumers, with a wider range of smart devices and connected equipment” [2]. The World Health Organization (WHO) has launched the Global Strategy on Digital Health 2020–2025, emphasizing that digital health should be an integral part of health priorities and should offer benefits to people, communities, and health services in an ethical, safe, and sustainable way. Therefore, the principles of transparency, accessibility, scalability, replicability, interoperability, security, and confidentiality are required [3].

The use of digital solutions in the field of preventive, curative, and health recovery services, but also for educational management in the field of health, management, and communication in health services, the development of health policies has the potential to bring benefits to patients, health professionals, and medical systems [4,5]. However, the development of this area faces many challenges regarding human resource training, design, testing, implementation, evaluation of solutions appropriate to the proposed purpose, the needs of the target group, dissemination of results and their inclusion in the structure of health systems, financial support and measures, and appropriate legislation [6,7,8].

In order to improve the digital health services in Romania, in 2022, a multiyear digital health partnership was built on a new memorandum of understanding signed by WHO/Europe and the Romanian Ministry of Health to provide concrete support as part of the country’s recovery and resilience plan [9].

Given that the ultimate role of medical training is to meet the medical needs of society in an optimal way, the purpose of current medical education must be to train skills in accordance with all available resources, including technological ones [10]. However, there is a considerable difference between the evolution of technological means in this field and the level of digital health education [11,12].

The need to incorporate training programs for the use of digital solutions in the education of medical staff is becoming increasingly recognized, and in recent years, there have been multiple initiatives in this regard in various medical centers or universities [13,14,15,16,17,18,19]. Medical students need a curriculum that enables them to critically analyze the options offered by information and communication technologies in their field, to implement them where they can be of use, and to be able to assess their effectiveness in applied contexts [7,8]. Incorporating the study of digital health into the curriculum of medical universities is seen as a challenge for most of them; there are a number of factors that should be taken into account. In addition to the digital competencies that provide a foundation in technical and computer literacy, digital health education needs to be constantly calibrated to the evolution of medical technologies and adapted to the requirements of practice [15,16,17]. Thus, the education programs in this field initiated during university study must be correlated with the concept of continuous medical training in order to ensure a permanent connection with the evolution of the technological instruments that appear [18,19].

Studies from several countries have started to investigate opinions and behaviors of medical students with regard to the use of digital health, as well as their needs and experiences regarding the training in this field, but until now no such study has been performed in Romania [14,15,16,17,18]. There are also studies which underline the need to investigate possible gender differences regarding students’ attitudes towards digital health and their desire for further training in this field, which contributes to the design of medical curricula to prepare all future physicians for the ongoing digitalization in the medical field in the most appropriate way [19].

Hence, this study focuses on medical students from the University of Medicine and Pharmacy in Cluj-Napoca, Romania, and has three objectives. First, it evaluates the opinions of medical students regarding their previous training as well as their needs for future training in the field of digital health. Second, it assesses their attitudes regarding digital health and their intention to use digital tools as physicians. Lastly, the interrelationship between these issues as well as the socio-demographic factors which influence them are investigated.

## 2. Materials and Methods

### 2.1. Study Sample and Procedure for Data Collection

A cross-sectional survey was performed during the COVID-19 pandemic (June–August 2021) among fifth and sixth year students of the Faculty of Medicine from the Iuliu Hațieganu University of Medicine and Pharmacy in Cluj-Napoca, Romania. It is part of a research study which received approval from the Ethic Commission of Iuliu Hatieganu University of Medicine and Pharmacy (Approval no. 98/5.04.2021).

All students in years 5 and 6 from the Romanian section of the faculty were invited to participate in the study by completing an online anonymous questionnaire. All students were informed that participation was voluntary and involved the filling in of an anonymous questionnaire; by filling in the questionnaire, students agreed to participate. Out of the 712 students who received the invitation, 306 participated and completed the online questionnaire (acceptance rate 50%).

### 2.2. Instrument for Data Collection

An anonymous questionnaire was developed for this study based on data from the literature [20,21,22,23].

The present study included data regarding socio-demographic characteristics (gender, year of study) as well as several issues related to the training in the field of digital health, opinions, and intention to use digital tools for the following three areas: (a) Health promotion, health education, and disease prevention. (b) Early detection of diseases and monitoring and encouragement of patients for the self-management of the diseases. (c) Diagnosis, treatment, rehabilitation in various diseases.

For each of these three areas, students were asked if they received training/practical examples in different departments/format during their medical education (possible answers were: To a high extent, To some extent, None, I do not remember), as well as to which extent they agree/disagree that digital tools could be useful for different domains of these areas and their intention to use digital tools in the future as physicians for each of these domains.

Moreover, the students were asked if they would like to receive more training with regard to digital health (possible answers were: Yes, No, I do not know), and if they think that a formal program regarding digital health should be included in the curricula of medical students (possible answers varied on a 4-point scale from I totally disagree to I totally agree).

### 2.3. Data Analyses

The prevalence of the investigated issues was calculated.

Several indexes were created:

- One index of training (index-previous training) by adding the scores regarding the training for use of digital tools for each of the three areas.
- Four indexes of attitudes: one index of attitudes for each of the three areas (index-attitudes 1 by adding the scores of attitudes regarding the use of digital tools for prevention, index-attitudes 2 by adding the scores of attitudes regarding the use of digital tools for early detection and self-management, and index-attitudes 3 by adding the scores of attitudes regarding diagnoses, treatment, and rehabilitation), as well as an index for all attitudes (index-attitudes total which was created by adding the previous three indexes together).
- Four indexes of intention to use digital tools as physicians in the future: one index of intention for each of the three areas (index-intentions 1 by adding the scores of intentions regarding the use of digital tools for prevention, index-intentions 2 by adding the scores of intentions regarding the use of digital tools for early detection and self-management, and index-intentions 3 by adding the scores of intentions regarding diagnoses, treatment, and rehabilitation), as well as an index for all attitudes (index-intentions total which was created by adding the previous three indexes together).

Univariate linear regression analyses were employed for a better understanding of the factors which influence the attitudes for each area (the dependent variables were the three indexes -index-attitudes 1, index-attitudes 2, and index-attitudes 3-, while the independent variables were gender, study year, and previous training in each area), as well as the intentions for each area (the dependent variables were the three indexes -index-intentions 1, index-intentions 2, and index-intentions 3-, while the independent variables were gender, study year, the index of attitudes for each area, and previous training in each area).

At the same time, univariate linear regression analyses were used to assess the factors which influenced the opinions of the students regarding their need for more training in the field of digital health, as well as their agreement for the need of a formal training in the medical curricula for this field (the independent variables were gender, study year, the index-attitudes total, the index-intentions total, and index-previous training).

Statistical analyses were performed using SPSS 22 statistical package, with statistical significance being considered at *p* < 0.05.

## 3. Results

### 3.1. Training in the Field of Digital Health

The final sample is represented by the 306 students who filled in the questionnaire (63.3% of the participants were women, the higher percentage of women being in accordance with the fact that more women than men are enrolled at the Faculty of Medicine, while 57.2% were students from the fifth year and the rest from the sixth year of study).

As presented in Table 1, between 45% and 48% of students declared that they benefited from training or different practical examples during medical education regarding the use of digital tools in the three medical areas investigated by this study, while the rest said they did not benefit from these or did not remember.

A percentage of 84.6% said that they would like to receive more training in the field of digital health, 4.9% said that they do not want more training, while 10.5% declared that they have not decided with regard to this issue.

Moreover, when asked if they agree with the introduction of a formal training in the medical curricula regarding digital health, 58.2% said that they totally agree with this, 32.4% declared partial agreement, 3.6% expressed their disagreement, and 5.8% were undecided.

### 3.2. Attitudes and Intentions Regarding Digital Health

Table 2 shows that the majority of the students strongly agree or partially agree with the role of digital tools for different issues in the field of prevention, and a higher percentage of total agreement (more than 60%) was recorded with regard to the use of digital tools for recording data about/monitoring health risk behaviors and health education for the prevention of different health risk behaviors, as well as for offering information and possibilities of enrollment in different health education programs and the dissemination of these programs. Around half are fully convinced about their role for personalized counseling for health promotion.

Regarding the role of digital tools for the early detection of diseases and monitoring and encouragement of patients for self-management of the disease, the students were generally less convinced about their importance in comparison with the previous area; however, between 60 and 85% still recognize their role. The lowest percentage was received by digital tools which help patients with early detection and management of diseases.

The majority of the students agree totally or partially with the role of digital tools for the diagnosis, treatment, and rehabilitation in various diseases; the highest percentage of total agreement (more than 80%) was recorded with regard to their use for medical diagnoses, keeping medical records, and communication between health care professionals.

Table 3 depicts the intention of students regarding the use or recommending the use of digital tools in different domains from the three investigated areas. It shows that the majority of the students intend to use or recommend different digital tools for most of the domains from the three investigated areas, and generally the higher percentages for total agreement are observed for those domains which also received the highest total agreement with regard to attitudes about their role and importance.

### 3.3. Interrelationship between Training, Attitudes, and Intentions and the Socio-Demographic Factors Associated with Them

Table 4 shows that women had stronger attitudes than men with regard to the role of digital tools for prevention, as well as diagnoses, treatment, and rehabilitation, but not for early detection and self-management, while no age differences were found with regard to these issues. On the other hand, stronger attitudes were recorded regarding the role of digital tools for prevention as well as early detection among those who declared that they benefited from education in these particular fields during their medical school studies.

As presented in Table 5, the intention to use digital tools for each of the three fields was similar both among women and men, as well as among students from both study years, except that 6th year students were less convinced about the fact that they will use these tools for early detection. Stronger attitudes were also associated with stronger intention to use digital tools in the future for all three fields, while previous training influenced the first two fields.

Table 6 shows that no gender or age differences were found with regard to the students’ preferences for more training in the field of digital health as well as for the existence of a formal training program in this field. On the other hand, both preferences were higher among those with higher attitudes and intentions scores, while previous training did not influence these two preferences.

## 4. Discussions

Our study focused on training, attitudes, and intentions to use digital tools among Romanian medical students from one university in Romania. The results show that less than half declared they benefited from training, and examples regarding the use of digital tools pertained to three areas: (a) Health promotion, health education, and disease prevention. (b) Early detection of diseases and monitoring and encouragement of patients for self-management of the diseases. (c) Diagnosis, treatment, and rehabilitation in various diseases.

A recent study aimed to assess the perception of medical students regarding e-health in several European countries [22]. The results show that more than half of the students considered their ability to use e-health tools to be poor or very poor, while 84.9% of them answered that this is caused by lack of training and found it necessary to add digital health training to the university curricula. The information related to e-health that they wanted to be included in the courses was about data management, ethics, legal frameworks, research and entrepreneurial opportunities, its role in public health and health systems, communication skills, and practical training. The justification for these answers was based on the desire of students to have the appropriate preparation for the expectations they will face in their future medical activity [22].

At the same time, in our study, high percentages of students are convinced about the role of digital tools in different domains from these three areas; it was noted that these attitudes were more favorable in some domains, while less strong for others. These might be in relation to previous personal experiences, training, or examples received during medical education or informal education, and social and media influences [17,18,19,23]. The COVID-19 pandemic has resulted in an increase in patient use of telehealth services and the implementation of reimbursement for them in Romania [11]. Following the COVID-19 pandemic, the topic of e-health, m-health, and telemedicine received a new wave of attention in many countries including Romania, and once again the importance of accelerating the technological process is highlighted, reiterating the idea that digital health skills are becoming essential for providing efficient and safe care, with some domains being more affected than others [23,24]. Stronger attitudes in favor of the use of digital tools for the three investigated areas were associated with the desire for more training/examples during medical education for two of the areas. The year of study did not influence the attitudes indexes, while gender differences were noticed, with women having higher attitudes indexes for two of the areas. All these issues underline the need to offer appropriate training and examples in order to help students understand and value the importance and possible use of digital tools for different domains, including those which are not as well-recognized by medical students.

Moreover, looking to the intention to use digital tools in the future, it was noted that, generally, the domains from the three areas where intentions were higher were similar to those receiving more favorable attitudes; all three intentions indexes were statistically significantly influenced by the respective attitudes index, and two of them were also influenced by the previous training in that area. No gender differences were identified, while the year of study influenced the intentions index from the field of early detection of diseases and monitoring and encouragement of patients for self-management of the disease, with older students having lower intentions.

The majority of the participating students, independent of gender or year of study, declared that they would like to receive more training in this field, and this desire was stronger among those with stronger attitudes and intentions. More than half also totally agreed that a formal training program should be included in the medical curricula. Studies from other countries also matched the finding that medical professionals are not prepared to make optimal use of the means offered by e-health. An important obstacle is the limited access to quality education for e-health and medical informatics, both for medical staff and those in university training. Paradoxically, although the accreditation requirements of the university curricula did not include digital health competencies, employers expected graduates to be able to use these technologies [15,16,17,25]. There is a need for collaboration between medical universities and medical organizations as well as the e-health industry in order to achieve an educational plan in line with the level of technological development found in medical practice [23]. Additionally, starting with the transformations imposed by the COVID-19 pandemic in several countries, including Romania, with the expansion of the use of remote monitoring devices in medicine, a necessary step is to include training for their optimal use and training among medical students. The current context provides an opportunity in this regard: instead of being blocked by the obstacles that often arise from the complexity of implementing a digital health training program, such as investing in the necessary technology and capacity to build human resources, universities should make efforts to assure appropriate training to familiarize students with the digital tools that have recently been integrated into many medical centers [24,25].

The limitations of this study are represented by the fact that it included only medical students from one university in Romania and, similar to other studies using online questionnaires, the response rate was 50%. Hence, the results cannot be generalized outside the sample.

## 5. Conclusions

To the best of our knowledge, this is the first study from Romania which investigated the training, attitudes, and intentions regarding the use of digital health among Romanian medical students. Similar to other studies from Europe and further afield, the results underline the need of medical students to receive more training in the field of digital health, including strong support for development of a formal program in this field during medical education. It also showed that students have positive attitudes and intentions to use digital tools in different domains of preventive and curative health care, which is once again in favor of the development of digitalization in health care and appropriate training for medical students and doctors to be able to take advantage of it, with benefits for medical professionals, patients, and medical systems.

## Figures and Tables

**Table 1:** Previous training/practical examples regarding the use of digital tools for different medical areas.

Areas	To a High Extent %	To Some Extent %	None %	I Do Not Remember %
Health promotion, health education, and disease prevention	5.9	41.5	40.2	12.4
Early detection of diseases and monitoring and encouragement of patients for self-management of the disease	4.2	40.5	42.5	12.7
Diagnosis, treatment, and rehabilitation in various diseases	5.1	42.7	39.5	12.7

**Table 2:** Attitudes regarding digital health.

Areas/Domains	I Totally Agree %	I Partially Agree %	I Do Not Know %	I Partially Disagree %	I Totally Disagree %
Health promotion, health education, and disease prevention					
Recording data about/monitoring health risk behaviors	70.9	23.2	3.3	1.6	1.0
Information and education to promote a healthy lifestyle and prevent/reduce health risk behaviors	72.5	23.9	2.3	1.3	0
Personalized counseling to promote a healthy lifestyle and prevent/reduce health risk behaviors performed by health professionals using information and communication technology	54.6	31.7	7.8	4.9	1.0
Personalized counseling to promote a healthy lifestyle and prevent/reduce risky behaviors for computer-assisted health and mobile applications	55.6	30.4	6.9	6.2	1.0
Offering information and facilitating enrollment and participation in various health promotion programs	67.0	26.5	5.6	1.0	0
Continuation and dissemination of health promotion programs	74.5	19.9	4.6	0.7	0.3
Early detection of diseases and monitoring and encouragement of patients for self-management of the disease					
Digital tools that interpret symptoms or signs identified by the patient at self-examination	19.6	39.6	19.0	18.0	3.9
Tools that calculate and interpret risk scores for different diseases (adapted for use by patients)	60.5	30.7	6.5	1.6	0.7
Tools that inform about screening methods in certain pathologies and ways to access screening programs	69.6	24.6	4.6	1.0	0.3
Tools for monitoring medical parameters outside medical institutions	59.2	27.8	8.8	3.6	0.7
Tools that facilitate the self-management by patients of their own diseases	47.4	29.7	13.4	5.6	3.9
Diagnosis, treatment, and rehabilitation in various diseases					
Remote medical consulting	59.8	30.7	7.2	2.3	0
Periodic communication with patients and organization of support groups	71.2	25.2	2.6	0.7	0.3
Tools for healthcare professionals who calculate and interpret indices scores	85.6	13.4	1.0	0.0	0.0
Tools for healthcare professionals that support the diagnostic decision	52.3	35.0	10.5	2.0	0.3
Tools for healthcare professionals which facilitate the establishment of a treatment plan	54.6	33.7	9.2	2.3	0.3
Tools for medical staff which keep track of patients (patient file in digital format)	84.6	11.8	2.9	0.7	0
Favoring communication between medical staff	85.0	12.4	1.6	1.0	0
Adequate training of medical staff	60.1	29.4	8.2	2.0	0.3
Remote medical rehabilitation programs	52.9	32.7	11.1	2.3	1.0
Tools that help patients with disabilities	73.9	18.6	5.2	2.0	0.3

**Table 3:** Intention to use digital tools in the future.

Areas/Domains	I Totally Agree %	I Partially Agree %	I Do Not Know %	I Partially Disagree %	I Totally Disagree %
Health promotion, health education and disease prevention					
Recording data about/monitoring health risk behaviors	75.2	18.0	4.2	1.6	1.0
Information and education to promote a healthy lifestyle and prevent/reduce health risk behaviors	74.8	20.3	3.6	0.7	0.7
Personalized counseling to promote a healthy lifestyle and prevent/reduce health risk behaviors performed by health professionals using information and communication technology	56.5	28.1	11.8	2.6	1.0
Personalized counseling to promote a healthy lifestyle and prevent/reduce risky behaviors for computer-assisted health and mobile applications	58.5	27.8	8.8	3.9	1.0
Offering information and facilitating enrollment and participation in various health promotion programs	71.6	22.2	3.9	1.0	1.3
Continuation and dissemination of health promotion programs	77.1	17.3	3.9	0.7	1.0
Early detection of diseases and monitoring and encouragement of patients for self-management of the disease					
Digital tools that interpret symptoms or signs identified by the patient at self-examination	24.8	31.4	20.3	15.0	8.5
Tools that calculate and interpret risk scores for different diseases (adapted for use by patients)	59.5	27.8	10.1	2.0	0.7
Tools that inform about screening methods in certain pathologies and ways to access screening programs	72.2	22.5	4.6	0.3	0.3
Tools for monitoring medical parameters outside medical institutions	60.8	28.4	7.2	2.6	1.0
Tools that facilitate the self-management by patients of their own diseases	53.3	26.1	11.8	5.9	2.9
Diagnosis, treatment, and rehabilitation in various diseases					
Remote medical consulting	57.5	32.7	6.5	2.6	0.7
Periodic communication with patients and organization of support groups	72.2	22.9	4.2	0.3	0.3
Tools for healthcare professionals, who calculate and interpret indices scores	81.0	15.4	2.6	0.7	0.3
Tools for healthcare professionals that support the diagnostic decision	54.9	32.0	9.8	2.3	1.0
Tools for healthcare professionals which facilitate the establishment of a treatment plan	58.5	30.1	8.5	2.3	0.7
Tools for medical staff which keep track of patients (patient file in digital format)	83.0	14.1	2.3	0.7	0.0
Favoring communication between medical staff	83.3	14.7	2.0	0.0	0.0
Adequate training of medical staff	64.4	23.9	9.5	1.6	0.7
Remote medical rehabilitation programs	56.5	29.4	11.8	1.0	1.3
Tools that help patients with disabilities	73.9	18.6	6.2	1.0	0.3

**Table 4:** Factors which influence attitudes—results of linear regression.

	Index-Attitudes 1 Standardized Beta (CI)	Index-Attitudes 2 Standardized Beta (CI)	Index-Attitudes 3 Standardized Beta (CI)
Gender	−0.116 (−1.555–0.17)	NS	−0.120 (−2.06–0.072)
Study year	NS	NS	NS
Previous training in the field of digital tools for prevention	0.199 (0.285–1)		
Previous training in the field of digital tools for early detection and self-management of diseases		0.146 (0.107–0.804)	
Previous training in the field of diagnoses, treatment, and rehabilitation			NS

NS—nonsignificant.

**Table 5:** Factors which influence intention to use digital tools in the future—results of linear regression.

	Index-Intentions 1 Standardized Beta (CI)	Index-Intentions 2 Standardized Beta (CI)	Index-Intentions 3 Standardized Beta (CI)
Gender	NS	NS	NS
Study year	NS	−0.135 (−1.623–−0.153)	NS
Index-attitudes 1	0.599 (0.562–0.762)		
Index-attitudes 2		0.765 (0.723–0.875)	0.869 (0.860–0.990)
Index-attitudes 3			
Previous training in the field of digital tools for prevention	0.113 (0.002–0.802)		
Previous training in the field of digital tools for early detection and self-management of diseases		0.187 (0.249–0.972)	
Previous training in the field of diagnoses, treatment, and rehabilitation			NS

NS—nonsignificant.

**Table 6:** Factors which influence opinions regarding the need for future training—results of linear regression.

	Opinion Regarding the Need for More Training in the Field of Digital Health Standardized Beta (CI)	Opinion Regarding the Introduction of a Formal Training in the Field of Digital Health Standardized Beta (CI)
Gender	NS	NS
Study year	NS	NS
Index-attitudes total	0.280 (0.011–0.024)	0.325 (0.020–0.040)
Index-intentions total	0.290 (0.010–0.022)	0.396 (0.024–0.042)
Index-previous training	NS	NS

NS—nonsignificant.

## Data Availability

Data can be obtained on justified cases from the corresponding author.
